# Positional relationship between lacrimal sac and skull base: implication of risk of cerebrospinal fluid leakage during dacryocystorhinostomy

**DOI:** 10.1038/s41598-022-18859-5

**Published:** 2022-08-24

**Authors:** Shinjiro Kono, Aric Vaidya, Munekazu Naito, Takashi Nakano, Makoto Ito, Hirohiko Kakizaki, Yasuhiro Takahashi

**Affiliations:** 1 Department of Oculoplastic, Orbital & Lacrimal Surgery, Aichi Medical University Hospital, 1-1 Yazako-Karimata, Nagakute, Aichi 480-1195 Japan; 2 Department of Oculoplastic, Orbital & Lacrimal Surgery, Kirtipur Eye Hospital, Kathmandu, Nepal; 3 Department of Anatomy, Aichi Medical University, Nagakute, Aichi Japan; 4 Department of Radiology, Aichi Medical University, Nagakute, Aichi Japan

**Keywords:** Anatomy, Risk factors

## Abstract

Cerebrospinal fluid (CSF) leakage is a rare but severe complication during dacryocystorhinostomy (DCR). Understanding the details of the anatomy of the lacrimal drainage system and skull base is essential to avoid this complication. We examined the positional relationship between the lacrimal sac and skull base using 16 cadavers (22 sides) and using computed tomographic images taken in 81 patients (81 sides). Consequently, the frontal sinus intervened between the lacrimal sac and skull base in 81.8–90.1% of cases. The lacrimal sac fundus and posterior lacrimal crest were far from the skull base/cribriform plate, and the skull base above the lacrimal sac was considerably thick. These results indicate that the risk of skull base injury and consequent CSF leakage during DCR is extremely low. However, surgeons should be cautious of this complication by indirect injury due to a twisting movement of a bone rongeur applied to the maxillary bone during creation of a bony window in patients with no interposition of the frontal and ethmoid sinuses between the lacrimal sac and skull base.

## Introduction

Cerebrospinal fluid (CSF) leakage is a rare but severe complication during dacryocystorhinostomy (DCR)^1–9^. The incidence of CSF leakage during DCR ranged from 0 to 0.04%^3,8^, and most literature on DCR-associated CSF leakage are case reports^4–9^. Possible etiologies of CSF leakage during DCR are direct and indirect injuries to the skull base^5^. Direct injury can occur when a bony window is excessively extended in the superior and posterior directions to the anterior part of the skull base^5^. Indirect injury can occur when a twisting movement of a bone rongeur is applied to the maxillary bone during the creation of a bony window, causing a spiral fracture involving the skull base^1,5^. The cribriform plate is a common part of injury during DCR because of its thinness and lower position^6^, and its position varies among individuals and races^8^. Surgeons should understand the details of the anatomy of the lacrimal drainage system and skull base, especially the cribriform plate, to avoid CSF leakage during DCR.

Previous anatomical studies examined the positional relationship between the lacrimal drainage system and the skull base^1,10,11^. Botek et al. measured the distance from the common canalicular orifice (CCO) to the cribriform plate, but they did not consider the frontal sinus lying between the lacrimal sac and skull base (frontal sinus interposition)^11^, which prevents the transmission of twisting and rotational forces from a rongeur to the skull base. Neuhaus et al. examined the distance from the margins of a bony window to the frontal sinus and cribriform plate^1^. However, they removed the whole lacrimal bone to create the bony window^1^. Complete exposure of the CCO is an important step of DCR to increase its success rate^12^. However, as the CCO is located considerably anterior to the posterior lacrimal crest, such a posteriorly extended bony window is not necessary. In addition, the previous 2 studies included only a small number of cadavers (10 sides from 10 cadavers^11^ and 6 sides from 3 cadavers^1^). Kurihashi et al. measured the vertical distances from the points 10 mm and 20 mm posterior to the medial canthus to the cribriform plate, respectively, on 28 sides of 28 cadavers^10^. However, the reason for chosing these reference points for the measurements was unclear.

In this study, we examined the positional relationship between the lacrimal sac, skull base including the cribriform plate, and frontal and ethmoid sinuses for the implication of CSF leakage during DCR using cadavers and computed tomographic (CT) images.

## Results

The raw data is included in the supplementary file.

### Experimental cadaveric study

The measurement results and statistical comparison are shown in Table 1. In this experimental study, 16 embalmed Japanese cadavers were used (22 sides; 7 males and 9 females; 12 right and 10 left; mean age at the time of death, 84.6 years).

**Table 1:** Demographic data on cadavers, measurement results, and statistical comparison in the experimental study.

		Total	Male	Female	P value	Interposition of frontal sinus above lacrimal sac		P value
						Presence	Absence	
Number of cadavers/sides		16/22	7/8	9/14		13/18	3/4	
Age at death (years)		84.6 ± 8.1	86.1 ± 4.7	83.4 ± 10.1	0.837	85.5 ± 8.4	81.0 ± 6.2	0.342
Sex (M/F)						6/7	1/2	1.000
Vertical width of MCT (mm)		3.2 ± 0.8	2.9 ± 1.0	3.3 ± 0.6	0.482	3.2 ± 0.7	3.3 ± 1.3	0.594
Distance from upper edge of MCT (mm)	CCO	− 0.9 ± 1.8	− 0.3 ± 1.9	− 1.3 ± 1.7	0.188	− 1.0 ± 1.7	− 0.7 ± 2.6	0.902
	Sac fundus	4.4 ± 2.1	5.5 ± 2.1	3.7 ± 1.9	0.059	4.1 ± 1.8	5.6 ± 3.3	0.386
	Skull base	14.0 ± 3.9	12.6 ± 1.9	14.8 ± 4.6	0.297	13.3 ± 3.5	16.9 ± 4.7	0.098
	Frontal sinus top	26.6 ± 5.7	26.3 ± 6.4	26.7 ± 5.5	0.659			
Thickness (mm)	Frontal bone at level of skull base	4.1 ± 1.1	3.6 ± 0.8	4.3 ± 1.2	0.179			
	Skull base	4.6 ± 2.9	3.7 ± 4.0	5.2 ± 2.1	0.212	3.7 ± 1.9	8.9 ± 3.3	0.002
	Frontal bone at level of frontal sinus top	8.6 ± 2.0	8.4 ± 2.7	8.7 ± 1.6	1.000			

M, male; F, female; MCT, medial canthal tendon; CCO, common canalicular orifice.

The mean vertical width of the medial canthal tendon (MCT) was 3.2 mm. The mean vertical distances of the CCO and lacrimal sac fundus from the superior edge of the MCT were -0.9 mm and 4.4 mm, respectively. The maximum distance from the CCO to the superior edge of the MCT was 2.6 mm. By subtraction of the MCT-fundus distance from the MCT-CCO distance, the mean distance from the CCO to the lacrimal sac fundus was 5.3 mm. The vertical distances of the skull base and frontal sinus top from the upper edge of the MCT were 14.0 mm and 26.6 mm, respectively. The mean thickness of the skull base was 4.6 mm, and the mean thicknesses of the frontal bone at the horizontal level of the skull base and that at the horizontal level of the top of the frontal sinus were 4.1 mm and 8.6 mm, respectively. There was not any sex-related difference in age or measurement results (P > 0.050).

The frontal sinus intervened between the lacrimal sac and the skull base on 18 sides (81.8%). The vertical distance between the superior edge of the MCT and skull base tended to be longer in cadavers without frontal sinus interposition (P = 0.098). The skull base was thicker in cadavers without frontal sinus interposition (P = 0.002). Cadaver age at death and the other measurement results were not significantly different between the groups (P > 0.050).

### CT study

The measurement results and statistical comparison are shown in Tables 2 and 3. Although 88 patients with a patent lacrimal passage on the unaffected side underwent unilateral DCR during the study period, 7 patients were excluded from this study because CT was not taken in 3 patients and slice thickness was more than 1 mm in 4 patients. This study included 81 sides from 81 patients (22 males and 59 females; unaffected sides, 41 right and 40 left; patient age, 66.4 years; range of patient age, 17 to 90 years).

**Table 2:** Demographic data on patients, measurement results, and sex-related comparison in the computed tomographic study.

		Total	Male	Female	P value
Number of patients		81	22	59	
Age (years)		66.4 ± 16.3	68.6 ± 15.4	65.6 ± 16.7	0.460
Anterior plane (mm)	Distance of sac fundus-skull base	17.8 ± 5.6	19.2 ± 4.3	17.3 ± 6.0	0.167
	Thickness of skull base	4.4 ± 3.0	4.1 ± 2.5	4.5 ± 3.2	0.579
Posterior plane (mm)	Anteroposterior distance	16.9 ± 7.0	18.7 ± 5.5	16.3 ± 7.4	0.173
	Distance of sac fundus-skull base	7.7 ± 3.2	7.2 ± 3.6	8.1 ± 3.3	0.306
	Vertical distance of sac fundus-cribriform plate	4.2 ± 3.7	3.4 ± 3.8	4.6 ± 3.6	0.188
	Horizontal distance of sac fundus-cribriform plate	7.7 ± 2.0	8.1 ± 2.2	7.5 ± 1.8	0.192
	Thickness of skull base	2.8 ± 1.5	2.9 ± 1.9	2.7 ± 1.4	0.713
	Thickness of cribriform plate	1.4 ± 0.7	1.3 ± 0.5	1.4 ± 0.7	0.612

**Table 3:** Comparison of demographic data and measurement results between patients with and without frontal sinus interposition and between patients with and without the cribriform plate positioned above the level of the lacrimal sac fundus.

		Frontal sinus interposition		P value	Cribriform plate		P value
		Presence	Absence		Above lacrimal sac fundus	Below lacrimal sac fundus	
Number of patients		73	8		63	10	
Age (years)		66.0 ± 16.9	69.3 ± 9.3	0.887	67.1 ± 16.9	59.6 ± 16.5	0.180
Sex (M/F)		21/52	1/7	0.437	16/47	5/5	0.139
Anterior plane (mm)	Distance of sac fundus-skull base	19.1 ± 3.7	5.8 ± 5.7	< 0.001	19.5 ± 3.7	17.1 ± 3.6	0.116
	Thickness of skull base	3.7 ± 1.8	11.0 ± 3.9	< 0.001	3.5 ± 1.7	5.0 ± 2.2	0.008
Posterior plane (mm)	Anteroposterior distance				15.7 ± 4.3	18.3 ± 6.9	0.373
	Distance of sac fundus-skull base				8.3 ± 2.9	3.6 ± 1.5	< 0.001
	Vertical distance of sac fundus- cribriform plate	4.1 ± 3.7	5.4 ± 3.2	0.261	5.0 ± 3.2	-1.5 ± 1.2	< 0.001
	Horizontal distance of sac fundus- cribriform plate	7.9 ± 1.8	5.8 ± 2.7	0.012	7.9 ± 1.8	8.0 ± 1.3	0.962
	Thickness of skull base				2.7 ± 1.6	2.9 ± 1.2	0.248
	Thickness of cribriform plate	1.3 ± 0.6	1.9 ± 0.8	0.049	1.3 ± 0.6	1.3 ± 0.6	0.676

M, male; F, female.

On the coronal plane showing the lacrimal sac fundus and top of the lacrimal sac fossa (anterior plane), the mean vertical distance from the lacrimal sac fundus to the skull base was 17.8 mm, and the mean thickness of the skull base was 4.4 mm. On the coronal plane showing no interposition of an ethmoid air cell above the ethmoidal notch^13^ (no ethmoid sinus interposition) (posterior plane), the anteroposterior distance of this plane from the posterior lacrimal crest was 16.9 mm. The minimum anteroposterior distance was 4.9 mm. We measured the vertical distance from the level of the lacrimal sac fundus to the skull base and the skull base thickness in patients with frontal sinus interposition. The mean vertical fundus-skull base distance was 7.7 mm. The mean vertical and horizontal distances of the level of the lacrimal sac fundus from the cribriform plate were 4.2 mm and 7.7 mm, respectively. The mean thicknesses of the skull base and cribriform plate were 2.8 mm and 1.4 mm, respectively. There was no sex-related difference in patient age and measurement results (P > 0.050).

The frontal sinus lied between the lacrimal sac fundus and skull base in 73 patients (90.1%). On the anterior plane, in patients without frontal sinus interposition, the vertical distance from the lacrimal sac fundus to the skull base was shorter (P < 0.001), and the skull base was thicker (P < 0.001). On the posterior plane, in patients without frontal sinus interposition, the horizontal distance between the level of the lacrimal sac fundus and the cribriform plate was shorter (P = 0.012), and the cribriform plate was thicker (P = 0.049). In addition, in patients without frontal sinus interposition, formation of the inferior boundary of the cribriform plate was found 7.6 mm posterior to the posterior lacrimal crest, which was shorter than the distance from the posterior lacrimal crest to the posterior plane in patients with frontal sinus interposition (17.9 mm; P < 0.001; Mann–Whitney U test). Patient age and the other measurement results were not significantly different between the patient groups (P > 0.050).

Among 73 patients with frontal sinus interposition, the cribriform plate hung down below the horizontal level of the lacrimal sac fundus in 10 patients (13.7%). The maximum negative vertical distance was − 3.52 mm. On the anterior plane, the skull base was thicker in patients with the cribriform plate positioned below the level of the lacrimal sac fundus (P = 0.008). On the posterior plane, the vertical distance from the horizontal level of the lacrimal sac fundus to the skull base was shorter in patients with the cribriform plate positioned below the level of the lacrimal sac fundus (P < 0.001). Patient age and the other measurement results were not significantly different between these patient groups (P > 0.050).

## Discussion

The positional relationship between the lacrimal sac and skull base was examined in this study. On the anterior plane, the frontal sinus lies between the lacrimal sac and skull base in most cases, which prevents transmission of twisting and rotational forces to the bone produced by movement of a rongeur and following indirect injury to the skull base. The skull base and top of the frontal sinus were considerably found to be away from the lacrimal sac fundus. Although the maxillary bone around the anterior lacrimal crest is considerably thick (the maximum thickness, 4.6–6.3 mm)^14,15^, the thickness of the skull base was similar. Furthermore, the frontal bone at the level of the top of the frontal sinus was thicker than the maxillary bone. The posterior plane was farther from the lacrimal sac fundus. The cribriform plate was positioned above the horizontal level of the lacrimal sac fundus in most cases. These results indicate that the risk of CSF leakage by both direct and indirect injuries to the skull base during creation of a bony window in DCR is extremely low.

Primary nasolacrimal duct obstruction and dacryocystitis are more common in females^16^, and they usually have a small face, which had proposed an expectation that the distance from the lacrimal sac fundus to the skull base is shorter in females. However, our study showed that there was no significant sex-related difference in the distance from the lacrimal sac fundus to the skull base, indicating that the risk of CSF leakage is not different between sexes.

Our study demonstrated that 9.9–18.2% of cases had no frontal sinus interposition. In such cases, the cribriform plate was anteroposteriorly and horizontally closer to the lacrimal sac fundus than those with frontal sinus interposition. In this situation, a superior and posterior extension of the creation of a bony window may cause direct injury to the skull base. However, the skull base was considerably far from the lacrimal sac fundus and was significantly thicker in cases without frontal sinus interposition. Furthermore, the cribriform plate formed the inferior boundary of the olfactory fossa 7.6 mm posterior to the posterior lacrimal crest. Therefore, direct injury of the skull base/cribriform plate is rarely expected. On the contrary, the twisting movement of a bone rongeur during bone removal can be transmitted to the skull base/cribriform plate in cases without frontal sinus interposition. Surgeons should avoid the twisting and rotational movements in cases without frontal sinus interposition to prevent indirect injuries to the skull base/cribriform plate.

Among patients with frontal sinus interposition, the cribriform plate was positioned below the horizontal level of the lacrimal sac fundus in 13.7% of patients. In such patients, the skull base was also closer to the horizontal level of the lacrimal sac fundus on the posterior plane. However, the distance from the posterior lacrimal crest to the posterior plane was 17.9 mm, indicating that direct injury of the skull base/cribriform plate is improbable.

Previous articles recommended using the MCT as a landmark for the creation of a bony window without skull base injury^3,10,12,17,18^. Our previous study demonstrated that among 75 orbits from 48 cadavers, the maximum distance between the CCO and superior edge of the MCT was 2.32 mm^12^. This result was similar to the one in our present study that the maximum distance between the CCO and superior edge of the MCT was 2.58 mm. As complete exposure of the CCO is an important step of DCR to increase the success rate of DCR^12^, these results imply that the bone removal till the level of around 3 mm above the MCT is necessary for complete exposure of the CCO. On the contrary, Kurihashi et al. found that on the coronal section through 10 mm posterior to the medial canthus, the skull base was positioned only 3 mm above the horizontal level of the medial canthus in 21.4% of cadavers^10^. Based on this result, several articles advocated that a bony window should not be extended 3 mm above the MCT^3,17,18^. Our present study also found that the cribriform plate was positioned below the horizontal level of the lacrimal sac fundus in several cases, and the maximum negative vertical distance was -3.52 mm. However, as a matter of course, the CCO was always located below the lacrimal sac fundus, and the mean distance from the CCO to the lacrimal sac fundus was 5.3 mm, indicating that when bone removal extends superiorly till the exposure of the CCO, the superior margin of the bony window does not usually reach the horizontal level of the cribriform plate.

The cribriform plate is thought to be located more inferiorly in Asians^8^. The study by Neuhaus et al. in Caucasians demonstrated that the mean vertical distance from the superior margin of a bony window to the cribriform plate at the level of the posterior limit of the frontal sinus was 5.7 mm^1^. Similarly, Botek et al. showed that the mean vertical distance from the CCO to the most anteroinferior part of the cribriform plate was 15.13 mm^11^. In our study, the mean vertical distance of the horizontal level of the lacrimal sac fundus from the cribriform plate was 4.2 mm. Although the reference points for measurements were different between these previous^1,11^ and our present studies, the Japanese seem to have a lower-positioned cribriform plate.

To avoid twisting and rotational forces to the rongeur, alternative use of a drill and an ultrasonic bone aspirator is useful^19,20^. For patients with a high risk of CSF leakage during DCR, such as pre-existing deformity and an anatomical abnormality of the skull base^5,6^, the use of an image-guided navigation system may be helpful to avoid this complication^21^.

If CSF leakage occurs during DCR, the location and size of the dural injury should be confirmed^3^. Fat tamponade, tissue fibrin glue, and bone wax are used to close the point of leakage^3^. Patients should be monitored postoperatively, and consultation with neurosurgeons and otorhinolaryngologists should be obtained as appropriate^3^.

As the MCT is not identified during endoscopic DCR, the findings of this study will be more helpful for external DCR.

There are a few limitations to note in this study. First, this study included only Japanese cadavers and patients. Since there are known racial differences in lacrimal and skull base anatomy^12^, the findings presented here may not be applicable to other races. Second, all the measurements were performed by a single examiner. Measurements by plural number of examiners might have obtained more accurate results. Third, direct measurement of the anteroposterior distance in cadavers may be more precise. Fourth, we did not examine the position of the agger nasi cell that is an important anatomical structure during endoscopic DCR. A previous study showed that half of the patients had the agger nasi cell located medial to the lacrimal sac^22^. In such cases, this cell should be opened before the osteotomy. However, it is enough to open only the anterior part of the cell. In this situation, the risk of skull base injury is less. We, therefore, did not use this as a reference of the measurements.

In conclusion, the positional relationship between the lacrimal sac and skull base was examined in this study. The results of our study indicate that the risk of CSF leakage during DCR is extremely low because of a high rate of interposition of the frontal and ethmoid sinuses, the lacrimal sac positioned far away from the skull base/cribriform plate, and a thick skull base. However, oculoplastic surgeons should be cautious of this complication due to indirect injury in patients with no interposition of the frontal and ethmoid sinuses. Hence, it is essential to confirm the existence of sinus interposition on CT images before performing DCR.

## Materials and methods

### Experimental cadaveric study

#### Ethics approval/consent to participate

All cadavers that were included in this study had given their written informed consent prior to the deaths, allowing their bodies to be donated to our university for the advancement of clinical science. The format of the approval was consistent with Japanese law involving the “Act on Body Donation for Medical and Dental Education”. All cadavers were donated and registered with the cadaveric service of Aichi Medical University. The methods used for securing human tissues were humane and complied with the tenets of the Declaration of Helsinki.

This study was approved by the Institutional Review Board (IRB) of Aichi Medical University Hospital (approval number, 2021-042) and followed the tenets of the 1964 Declaration of Helsinki. At the request of the IRB, an outline of this study available for public viewing on the Aichi Medical University website was published. This public posting also gave the donors’ families an opportunity to decline participation in this study, although no refusal was made known to us.

#### Study design and cadavers

This experimental, anatomical study included cadavers fixed in 10% buffered formalin. None of the cadavers had any gross eyelid, orbital, lacrimal, cranial, paranasal, or facial disorders, or any history of eyelid, orbital, lacrimal, cranial, paranasal, or facial surgery while alive. Sides on which ethmoidectomy or skull base surgery was performed for another experimental study were excluded from this study.

#### Cadaver dissection and measurements

All the cadaver dissections and measurements were done by one of the authors (Y.T.). All the measurements were performed using a digital caliper (DigitalCaliper 19975; Shinwa Rules CO. Ltd., Niigata, Japan). Photographs were taken with a digital camera (D3100; Nikon, Tokyo, Japan).

**Figure 1:**
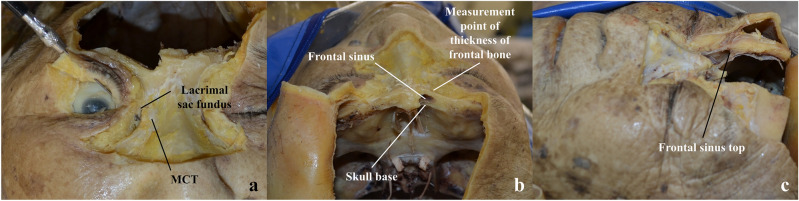
Cadaveric dissection. (**a**) A photo of a right orbit was taken from the front. The medial canthal tendon (MCT) and lacrimal sac fundus were exposed, and a probe was inserted from the lacrimal punctum to confirm the position of the common canalicular orifice. The frontal bone was removed up to the level of the skull base. (**b**) A photo of a right orbit was taken from the above. The frontal sinus lies between the lacrimal sac fundus and skull base. (**c**) A photo of a right orbit was taken from the left. The frontal sinus top at the vertical level of the lacrimal sac fundus was exposed.

The skin and orbicularis oculi muscle were removed until the MCT and lacrimal sac were exposed. After opening the lacrimal sac, a probe was inserted from the lacrimal punctum to confirm the position of the CCO (Fig. 1a). The cranial bone was partially removed up to the horizontal level of the skull base above the lacrimal sac (Fig. 1a). The presence or absence of frontal sinus interposition was confirmed (Fig. 1b). The vertical width of the MCT was measured. The vertical distances from the upper edge of the MCT to the CCO, lacrimal sac fundus, skull base, and the top of the frontal sinus were measured (Fig. 1a–c). The thicknesses of the skull base and frontal bone at the horizontal level of the skull base and that at the top of the frontal sinus were measured, respectively (Fig. 1b,c).

### CT study

#### Ethics approval/consent to participate

This study was approved by the IRB of Aichi Medical University Hospital (approval number, 2021-372) and followed the tenets of the 1964 Declaration of Helsinki. The IRB granted a waiver of informed consent for this study based on the ethical guidelines for medical and health research involving human subjects established by the Japanese Ministry of Education, Culture, Sports, Science, and Technology and the Ministry of Health, Labour, and Welfare. The waiver was granted because the study was a retrospective chart review, not an interventional study and because it was difficult to obtain consent from patients who had been treated several years prior to the study. Nevertheless, at the request of the IRB, we published an outline of the study, which is available for public viewing on the Aichi Medical University Hospital website. This public posting also allowed patients the opportunity to decline participation, although none of the patients did so. Personal identifiers were removed from all records before data analysis.

#### Study design and patients

This retrospective, observational study included all patients in whom CT was taken for differential diagnosis of lacrimal sac tumors before DCR for unilateral primary nasolacrimal duct obstruction +/− dacryocystitis between January 2018 and July 2021. Patients with missing data were excluded from this study. Patients in whom slice thickness of CT was more than 1 mm were also excluded from this study.

#### Data collection

The data on age, sex, affected side, and clinical diagnosis was collected from the medical charts of all the patients. Patency of the lacrimal drainage system on the unaffected side checked by lacrimal syringing was also confirmed from the medical records of all the patients.

#### Measurements on CT images

Continuous 1 mm axial CT images (Aquilion Precision; Canon, Tokyo, Japan) were obtained using a bone window algorithm (width, 2500; level, 500). Axial CT images were obtained based on Reid’s baseline, and coronal images were reconstructed based on planes parallel to the face. To obtain symmetric axial images, the positions of the face, shoulders, and soles of the feet were fixed with no bend in the patient’s neck. All measurements were performed on the unaffected side using the digital caliper tool of the image viewing software (ShadeQuest/ViewR; Yokogawa Medical Solutions Corporation, Tokyo, Japan) and that of the 3-dimensional (3D) image processing system (Aquarius NET; TeraRecon Incorporated, NC, US) by one of the authors (Y.T.).

**Figure 2:**
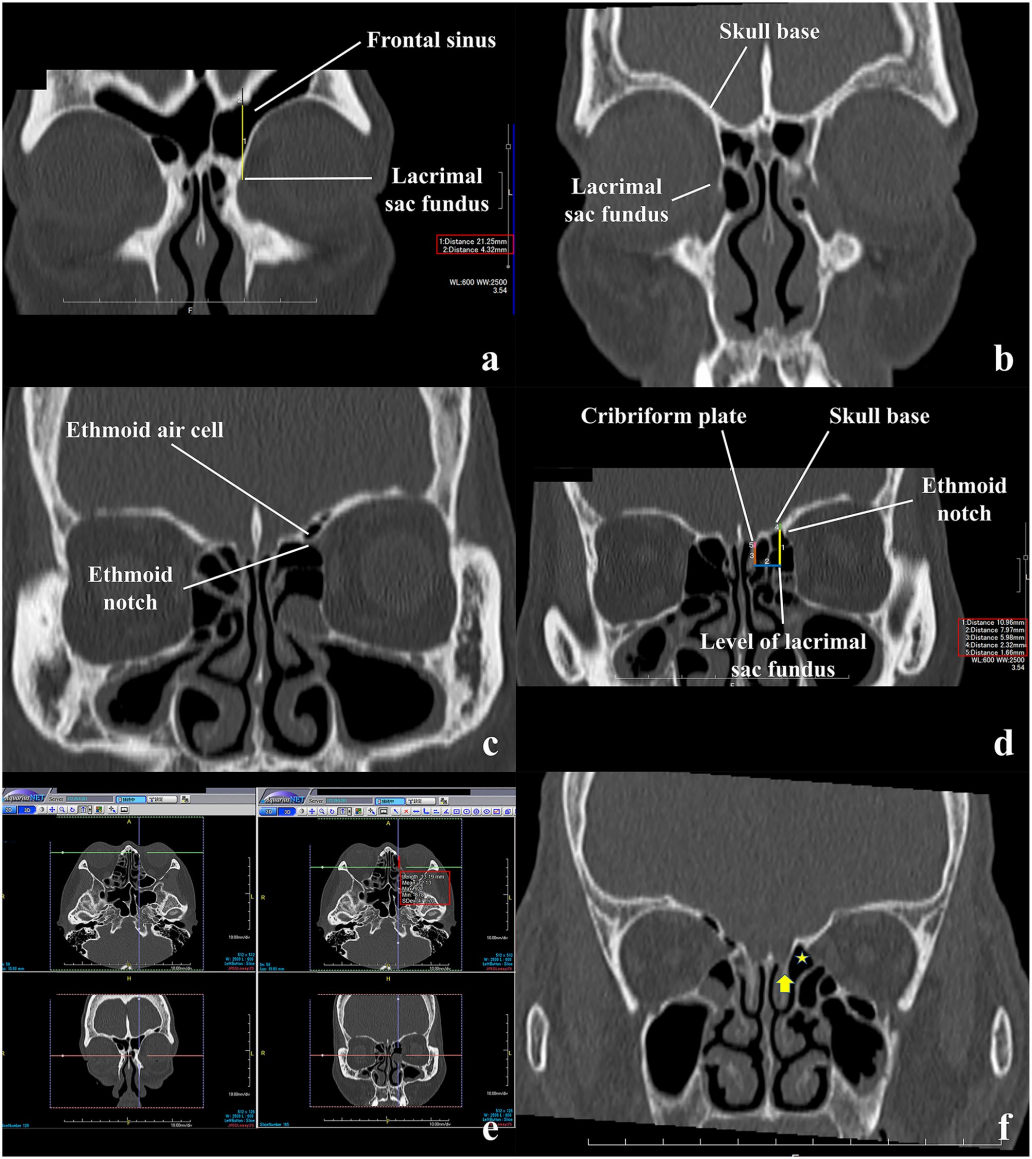
Measurements on computed tomographic (CT) images. The measurement results are surrounded by the red rectangles. (**a**,**b**) The coronal CT images showing the lacrimal sac fundus (anterior planes). Measurements of the distance between the lacrimal sac fundus and skull base (#1, yellow line) and the thickness of the skull base (#2, black line) in a patient with interposition of the frontal sinus between the lacrimal sac fundus and skull base (frontal sinus interposition) (**a**). No frontal sinus interposition (**b**). (**c**,**d**) The coronal CT images with (**c**) and without interposition of an ethmoid air cell above the ethmoid notch (ethmoid sinus interposition) (**d**, posterior plane). The vertical distance from the level of the lacrimal sac fundus to the skull base (#1, yellow line), the vertical (#3, brown line) and horizontal (#2, blue line) distances from the level of the lacrimal sac fundus to the lateral margin of the cribriform plate, thickness of the skull base at the vertical level of the lacrimal sac fundus (#4, green line), and that of the cribriform plate were measured (#5, pink line) (**d**). (**e**) First, the intersection of the cursor was fixed at the lacrimal sac fundus (left-bottom). The coronal section moved posteriorly until the section showing no ethmoid sinus interposition (right-bottom, posterior plane). The anteroposterior distance of the posterior lacrimal crest from the posterior plane was measured (red line) (right-upper). (**f**) The cribriform plate (arrow) is positioned below the horizontal level of the lacrimal sac fundus (asterisk).

On the anterior plane, the presence or absence of frontal sinus interposition was confirmed (Fig. 2a,b). The vertical distance between the lacrimal sac fundus and the skull base and the thickness of the bone of the skull base were measured (Fig. 2a). Next, the cursor was positioned and fixed at the lacrimal sac fundus, and coronal section images moved posteriorly to the posterior plane (Fig. 2c,d)^13^. On the posterior plane, the vertical distance from the cursor position to the skull base, the horizontal distance from the vertical line through the cursor to the cribriform plate, the vertical distance from the horizontal line through the cursor position to the cribriform plate, thicknesses of the bone of the skull base, and that of the cribriform plate at the lateral margin were measured (Fig. 2d).

Using the 3D image processing system, the intersection of the cursor was positioned and fixed at the lacrimal sac fundus on the anterior plane, and coronal section images moved posteriorly to the image showing no ethmoid sinus interposition. In this situation, the anteroposterior distance from the posterior lacrimal crest to the cursor position was measured on the axial image (Fig. 2e). In patients without frontal sinus interposition, the anteroposterior distance from the posterior lacrimal crest to the point where the cribriform plate formed an inferior boundary of the olfactory fossa was measured.

### Statistical analysis

The patient’s age and measurement results were expressed as means ± standard deviations. The vertical distances were expressed as positive values when the measurement points were above the reference points (the superior edge of the MCT and lacrimal sac fundus). The sex-related differences in the cadaver’s age at the time of death, patient’s age, and measurement results were analyzed using the Student’s t-test or Mann–Whitney U test. The cadaver’s age at the time of death, patient’s age, the ratio of sex, and measurement results were compared between cadavers/patients with and without frontal sinus interposition of the frontal sinus using the Mann–Whitney U test and Fisher’s exact test. Patient’s age, the ratio of sex, and measurement results were compared between patients with and without the cribriform plate positioned above the horizontal level of the lacrimal sac fundus (Fig. 2f) using the Mann–Whitney U test and Fisher’s exact test. All statistical analyses were performed using SPSS™ ver. 26 software (IBM Japan, Tokyo, Japan). A P*-*value of < 0.050 was considered statistically significant.

### Conference presentation

A part of this study was presented at the 36th Asia–Pacific Academy of Ophthalmology (APAO) Congress held during September 5–11, 2021.

## Supplementary Information

Supplementary Information.

## Data Availability

We submit a supplemental file including all data.
